# Significant association between ERCC2 and MTHR polymorphisms and breast cancer susceptibility in Moroccan population: genotype and haplotype analysis in a case-control study

**DOI:** 10.1186/s12885-018-4214-z

**Published:** 2018-03-15

**Authors:** Hanaa Hardi, Rahma Melki, Zouhour Boughaleb, Tijani El Harroudi, Souria Aissaoui, Noureddine Boukhatem

**Affiliations:** 1 Laboratory of Physiology, Genetics and Ethnopharmacology, Department of Biology, Faculty of Sciences, University of Mohammed First, Oujda, Morocco; 2 Oncology Center Mohamed VI Ibn Rochd, Casablanca, Morocco; 3 Faculty of Medecine, University of Mohammed First, Oujda, Morocco; 4 Hospices Civiles de Lyon, Lyon, France

**Keywords:** Breast cancer, ERCC2, MTHFR, Genetics, Single nucleotide polymorphism, Haplotype, Moroccan population

## Abstract

**Background:**

Genetic determinants of breast cancer (BC) remained largely unknown in the majority of Moroccan patients. The purpose of this study was to explore the association of *ERCC2* and *MTHFR* polymorphisms with genetic susceptibility to breast cancer in Moroccan population.

**Methods:**

We genotyped ERCC2 polymorphisms (rs1799793 (G934A) and rs13181 (A2251C)) and MTHFR polymorphisms (rs1801133 (C677T) and rs1801131 (A1298C)) using TaqMan SNP Genotyping Assays. Genotypes were compared in 151 BC cases and 156 population-matched controls. Allelic, genotypic and haplotype associations with the risk and clinicopathological features of BC were assessed using logistic regression analyses.

**Results:**

*ERCC2-*rs1799793-AA genotype was associated with high risk of BC compared to wild type genotype (recessive model: OR: 2.90, 95% CI: 1.34–6.26, *p* = 0.0069) even after Bonferroni correction (*p* < 0,0125). *MTHFR* rs1801133-TT genotype was associated with increased risk of BC (recessive model, OR: 2.49, 95% CI: 1.17–5.29, *p* = 0.017) but the association turned insignificant after Bonferroni correction. For the rest of SNPs, no statistical associations to BC risk were detected.

Significant association with clinical features was detected for *MTHFR*-rs1801133-TC genotype with early age at diagnosis and familial BC. Following Bonferroni correction, only association with familial BC remained significant. *MTHFR*-rs1801131-CC genotype was associated with sporadic BC. *ERCC2-*rs1799793-AA genotype correlated with ER+ and PR+ breast cancer. *ERCC2*-rs13181-CA genotype was significantly associated large tumors (*T* ≥ 3) in BC patients. None of these associations passed Bonferroni correction.

Haplotype analysis showed that *ERCC2* A-C haplotype was significantly associated with increased BC risk (OR: 3.71, 95% CI: 1.7–8.12, *p* = 0.0002 and *p* = 0.0008 before and after Bonferroni correction, respectively) and positive expression of ER and PR in BC patients. *ERCC2* G-C haplotype was correlated with PR negative and larger tumor (T4). We did not find any MTHFR haplotypes associated with BC susceptibility. However, the less common haplotype MTHFR T-C was more frequent in young patients and in familial breast cancer, while MTHFR C-C haplotype was associated with sporadic BC form.

**Conclusions:**

Our findings are a first observation of association between ERCC2 SNPs and breast cancer in Moroccan population. The results suggested that *ERCC2* and *MTHFR* polymorphisms may be reliable for assessing risk and prognosis of BC in Moroccan population.

## Background

Breast cancer (BC) is by far the most frequently diagnosed malignancy among women worldwide. It is a major public health problem in both developed and developing countries. The incidence of BC is steadily increasing over the years. For 2012, there were 1.7 million estimated new cases of BC [1]. More new cases occurred in less developed (883,000 cases) than more developed countries (794,000 cases) [1]. The growing trend has been reported by the Global Burden of Disease (GBD) study in 2015 covering 32 cancer groups in 195 countries. This study ranked the BC as the most common incident cancer for women (2.4 million cases), and as the leading cause of women cancer death (523,000 deaths) [2]. The BC incidence rates are geographically variable with higher rates occurring in Europe and North America than Africa and Asia. The incidence is constantly growing in the Arab countries while remaining below that recorded in Europe or in America [3].

Breast cancer has become the leading cause of malignancy in Moroccan females. The most recent data in Morocco (country of North-western Africa) have described an increasing incidence rate of breast cancer from 39.0 to 49.5 per 100,000 women between 2008 and 2012 [4]. This rate was relatively higher than in other regional countries, but it remained well below the incidence found in Western countries [5].

Breast cancer is a complex and heterogeneous multifactorial disease which is strongly influenced by environmental, lifestyle and genetics risk factors. The effect of rare highly and moderately penetrant alleles located in predisposition genes such as *BRCA1*, *BRCA2*, *TP53* and DNA repair genes explains only a small percentage of genetic risk of BC. To date, and through multiple previous genome-wide association studies (GWAS), large scale replication studies and meta-analysis studies, more than 90 breast cancer risk SNPs (single nucleotide polymorphisms) have been identified [6–11]. Although, individually, these common variants present relatively small increments in BC risk and a modest effect, taken together they may account for about 15–20% of familial clustering and a substantial proportion of sporadic BC susceptibility [12].

The study reported here concerned the population of Morocco. This country is located in the northwestern corner of the African continent (33°, 35’N latitude and 7°, 39’W longitude), bordered by the Mediterranean Sea to the north, the Atlantic Ocean to the west, Algeria to the east and Mauritania to the south. Morocco is host to a number of human populations that are different in their language, culture and ethnic identity. Indeed, this country very coveted since antiquity, and continues to attract peoples coming from the Mediterranean, the near and the Middle East, as well as from sub-Saharan Africa. The overwhelming majority of Moroccan population is composed of Berbers and Arabs. The Berbers, a people of Euro-Asiatic origin are indigenous residents of Morocco since at least 5000 years ago. They were invaded by many civilizations such as Phoenicians, Carthaginians, Romans, Vandals, Byzantines and Arabs. The Arabs came from the Middle East, namely from the Arabian Peninsula, in the 7th Century and conquered the country during the Islamic expansion in North Africa. Other human groups in Morocco are the Africans, Sub-Sahara Africans, Europeans (commonly descended from Spanish or French ancestry), and Sephardic Jews. All of these populations probably have contributed to the genetic diversity of the current population of Morocco.

The genetic basis of BC remains unknown in the majority of Moroccan patients. Identifying genetic factors associated with this prevalent disease is nevertheless of considerable clinical importance. To date, the few genetic studies that have been conducted in Moroccan population have demonstrated an important but complex contribution of genetic factors in BC pathogenesis as reflected by an increased frequency (27.5–31.6%) of *BRCA1/2* mutations detected in familial BC cases [13, 14]. Beside *BRCA1/2* pathogenic mutations, relatively few single nucleotide polymorphisms (SNPs) have been studied in Moroccan population. Investigations have yielded a small number of suggestive SNPs associated with varying risks of developing BC [15, 16].

Considered collectively, these observations strongly suggested that other loci may be involved in genetic predisposition to BC in Morocco and clear remaining hereditary BC risk. In the present study, we aimed to investigate the role of four common genetic variants (SNPs) in mediating the disease in Moroccan patients. All of them have been associated with BC in different populations. Their positive correlation with BC have been supported by a meta-analysis of 150 published meta-analysis studies grouping 4474 studies for various types of cancers, 2,452,510 cases and 3,091,626 controls [10].

The selected variants rs1799793 and rs13181 are linked to *ERCC2* gene (Excision repair cross-complementation group 2) and rs1801131, rs1801133 polymorphisms to *MTHFR* gene (methylenetetrahydrofolate reductase). These genes are involved in the etiology of cancer through crucial cellular pathways including, DNA repair path (*ERCC2*) [17], methylation and DNA synthesis (*MTHFR*) [18].

Herein, we examined the association between these SNPs and BC as well their contribution in modulating major breast cancer clinicopathological traits in Moroccan patients. Moreover, the effect of haplotypes formed by SNPs localized in the same gene was also examined. It should be mentioned that three selected SNPs were considered for the first time in our study in Moroccan BC cases. The MTHFR-rs1801133 polymorphism has been studied before on a group of 96 Moroccan patients [19]. The notable strength of our study, regarding this SNP, was to analyze a large number of patients from a different geographic area of Morocco and to test haplotype associations of *MTHFR* SNPs.

## Methods

### Study subjects

A total of 151 pathologically confirmed female breast cancer patients admitted to the Hassan II Regional Oncology Center of Oujda city during 2009–2013 were included in this study. This center covers the entire eastern region of Morocco in term of cancer diagnosis and patients management. The control group consisted of 156 age-matched healthy female with no prior history of any type of cancer, and who were recruited as volunteer blood donors at the Blood Transfusion Center of the same region. All cases and controls were genetically unrelated Moroccans from the same geographical area and were recruited during the same period.

Relevant clinicopathological characteristics recorded for each case were collected by review of patients’ medical files. The recorded information included age at diagnosis, family history of breast cancer, laterality, histology type, Scarff-Bloom-Richardson (SBR) grade, tumor size, lymph node involvement, metastases as well as hormone receptor status including: estrogen receptor (ER), progesterone receptor (PR), and human epidermal growth factor receptor 2 (Her-2).

The following inclusion criteria were used to identify family history (FH) breast cancer cases: single breast cancer diagnosed before the age of 39 years and/or bilaterality; three or more first or second degree relatives with breast cancer in the same side of the family tree; two first degree relatives with breast cancer, with at least one early onset breast cancer case (≤40 years) or male breast cancer case or ovarian cancer case; triple-negative breast cancer diagnosed before 50 years regardless to family history or > 50 years with positive family history of breast cancer; multiple primary cancers in the same individual or in the family. A case was considered sporadic in the absence of the above criteria.

The study protocol was reviewed and approved by the ethics committee of Mohammed VI University Hospital in Marrakech.

Before enrollment in the study and after explaining the procedures, written informed consent for research participation was signed by each participant.

Peripheral venous blood samples were collected from case and control groups into sterile EDTA coated tubes. Genomic DNA extraction was done using a standard salting-out method [20]. The isolated DNA samples were quantitated using the NanoVue Plus™ spectrophotometer (biochrom, Harvard Bioscience Inc. Massachusetts, USA), and stored at − 20 °C until analysis.

### Selection of SNPs and TaqMan genotyping

After a review of published literature, we selected four candidate SNPs suggested as significant risk factors for the breast cancer [10], specifically, rs1799793, rs13181 on *ERCC2* gene and rs1801133, rs1801131 on *MTHFR* gene.

Genotyping of the SNPs was performed by allelic discrimination using the TaqMan SNP Genotyping Assays according to the manufacturer’s instructions (Applied Biosystems). Specific primers and FAM/VIC – labeled TaqMan probes were designed and supplied by Applied Biosystems. Briefly, the reaction was performed in a 10 μl final volume containing 5 ng of genomic DNA, 1X TaqMan SNP Genotyping Assay and 1X TaqMan Genotyping master mix (that contains AmpliTaq Gold^®^ DNA Polymerase UP (Ultra Pure), dNTPs without dUTP, and passive internal reference based on proprietary ROX™ dye). All assays were carried out in 24-well plates including positive and negative controls. The PCR conditions were as follows: Initiation at 95 °C for 7 min, followed by 50 cycles of denaturation at 95 °C for 50 s and annealing/extension at 60 °C for 30s. Plates were read on a Thermo Scientific™ PikoReal™ Real-Time PCR System (Thermo Fisher Scientific Oy, Finland), and the alleles were assigned using the PikoReal Software v 2.2.

### Statistical analysis

Agreement of genotype frequencies to Hardy-Weinberg expectations was assessed independently among control and case groups for each SNP using the χ^2^ test analysis with one degree of freedom. Student’s *t-*test was used to evaluate the differences in mean age at diagnosis between the cases and controls.

The analysis of association between a single variant and breast cancer risk in multiple inheritance models (genotype, dominant, recessive, and additive) was presented in odds ratios (OR) with corresponding 95% confidence intervals (95% CI) using logistic regression. The allelic frequencies of each SNP were also compared between cases and controls. The wide-type genotype was regarded as the reference group. Logistic regression analyses restricted to case group were also performed to compute the odds ratio associating different genotypes with patients’ clinicopathological features.

Data management and statistical analyses were performed using the statistical package SPSS (version 21.0; IBM Corp. Armonk, NY, USA) and SNPStats software ().

Haplotype analysis was restricted to polymorphisms located on the same chromosome: the haplotype rs1801133-rs1801131 (*MTHFR*), and the haplotype rs1799793-rs13181 (*ERCC2*). Haplotype frequency distributions were deduced from genotype data and compared between cases and controls using UNPHASED software version 3.1.7 [21] as well as SNPStats program [22]. The most common haplotype was selected as the reference. Odds ratios and 95% CI were calculated to estimate the degree of the association between haplotypes and the risk of breast cancer.

Measure of linkage disequilibrium (LD) between each pair of SNPs, including Lewontin’s standardized disequilibrium coefficient (D’) and the squared correlation coefficient (r^2^), was computed with Haploview software package version 4.2 [23]. Results were confirmed using UNPHASED and SNPStats programs.

The effect of representative haplotypes on subphenotypes in breast cancer cases was assessed by SNPstats program, and results were presented as odds ratios with 95% CI.

A two-tailed *p* value less than 0.05 was taken as statistically significant. *P* values obtained were corrected for multiple testing using Bonferroni correction for the number of tests.

## Results

### Patient’s clinicopathological characteristics

The clinical characteristics of the breast cancer patients enrolled in this study were summarized in Table 1. The mean age of case and control groups at the time of diagnosis was 43.81 ± 9.74 years, (median = 43 years, range, 26–72 years) and 42.72 ± 9.29 years, (median = 41 years, range, 26–60 years), respectively. No statistically significant difference was observed in term of age between the two groups (*p* = 0.316).

**Table 1:** Clinicopathological features of the breast cancer patients included in the study

Variables	Cases (*n* = 151)		Controls (*n* = 156)		*P* value
	*n*	(%)	*n*	(%)	
Age at diagnosis					
Mean (years ± SD)	43.81 ± 9.74		42.72 ± 9.29		0.316
≤ 40	56	37.09	72	46.15	0.107
> 40	95	62.91	84	53.85	
Family history of breast cancer					
Sporadic	91	60.3			
Familial	60	39.7			
Laterality					
Bilateral	4	2.65			
Unilateral	147	97.35			
Histological type					
Ductal invasive	130	86.1			
Lobular invasive	16	10.6			
Others	5	3.3			
Histological grade					
I	10	6.6			
II	108	71.5			
III	27	17.9			
unknown	6	4			
Tumor size					
T1	24	15.9			
T2	74	49			
T3	16	10.6			
T4	12	7.9			
TX	8	5.3			
Unknown	17	11.3			
Lymph nodes status					
N0	48	31.8			
N+	92	60.9			
Unknown	11	7.3			
Metastasis status					
M0	127	84.1			
M+	19	12.6			
Unknown	5	3.3			
Her-2 status					
Positive	26	17.2			
Negative	113	74.8			
Unknown	12	7.9			
PR status					
Positive	89	58.9			
Negative	48	31.8			
Unknown	14	9.3			
ER status					
Positive	107	70.9			
Negative	36	23.8			
Unknown	8	5.3			
TN breast cancer	22	14.6			

*n* number of individuals (except for mean ± SD age), *%* percentage of individuals, *SD* standard deviation, *Her-2* Human epidermal growth factor receptor-2, *PR* progesterone receptor, *ER* estrogen receptor, *TN* triple negative

Out of 151 cases, 39.7% of them had a family history of breast cancer. Almost all patients (97.35%) had unilateral tumor. A large proportion have invasive ductal carcinoma (86.1%), and 71.5% had early stage tumor (Stage T2), while 13.2% had advanced-stage tumor (T3 and T4). Histopathologically, 71.5% of patients had intermediate grade II, and 17.9% presented high grade (grade III). Additionally, 60.9% exhibited lymph node involvement, while metastasis was confirmed in only 12.6% of cases. Regarding hormone receptor expression, most of cases harbored a Her-2 negative (74.8%), ER-positive (70.9%) and PR-positive (58.9%) tumor. Triple negative status was observed in 14.6% of patients.

### Associations between SNPs and breast cancer risk

A total of 156 controls and 151 BC subjects were successfully genotyped for the following selected SNPs: *ERCC2-*rs1799793 (G934A), *ERCC2-*rs13181 (A2251C), *MTHFR*-rs1801133 (C677T) and *MTHFR*-rs1801131 (A1298C).

Genotypes and alleles distributions of the 4 polymorphisms in BC case and control groups are depicted in Table 2. The genotype frequencies of all SNPs were in compliance with Hardy-Weinberg equilibrium in control group (*p* > 0.05). In patients group, the distribution of *ERCC2* rs1799793, *ERCC2* rs13181 and *MTHFR* rs1801133 genotypes did not conform to the HWE (*p* = 0.002, *p* = 0.007 and *p* = 0.013, respectively).

**Table 2:** Genotype and allele distribution of *ERCC2* and *MTHFR* polymorphisms in breast cancer cases and controls

SNP *(gene)*	Genotype	Cases (*n*, %)	Controls (*n*, %)	OR (95% CI)^a^	*p* ^b^
rs1799793 *(ERCC2)*					
Codominant	G/G	76 (50.3)	81 (51.9)	1	
Heterozygote	G/A	50 (33.1)	65 (41.7)	0.82 (0.51–1.33)	0.42
Homozygote	A/A	25 (16.6)	10 (6.4)	**2.66 (1.20–5.91)**	**0.016**
Dominant	G/G	76 (50.3)	81 (51.9)	1	0.78
	G/A-A/A	75 (49.7)	75 (48.1)	1.07 (0.68–1.67)	
Recessive	G/G-G/A	126 (83.4)	146 (93.6)	1	**0.0069**
	A/A	25 (16.6)	10 (6.4)	**2.90 (1.34–6.26)**	
Overdominant	G/G-A/A	101 (66.9)	91 (58.3)	1	0.12
	G/A	50 (33.1)	65 (41.7)	0.69 (0.44–1.10)	
Log-additive	–	–	–	1.29 (0.93–1.79)	0.13
Allele	G	202 (67)	227 (73)	1	0.11
	A	100 (33)	85 (27)	1.32 (0.94–1.87)	
HWE		*p* = 0.002	*p* = 0.52		
rs13181 *(ERCC2)*					
Codominant	A/A	80 (53)	91 (58.3)	1	
Heterozygote	C/A	50 (33.1)	52 (33.3)	1.09 (0.67–1.79)	0.72
Homozygote	C/C	21 (13.9)	13 (8.3)	1.84 (0.86–3.91)	0.11
Dominant	A/A	80 (53)	91 (58.3)	1	0.34
	C/A-C/C	71 (47)	65 (41.7)	1.24 (0.79–1.95)	
Recessive	A/A-C/A	130 (86.1)	143 (91.7)	1	0.12
	C/C	21 (13.9)	13 (8.3)	1.78 (0.86–3.69)	
Overdominant	A/A-C/C	101 (66.9)	104 (66.7)	1	0.97
	C/A	50 (33.1)	52 (33.3)	0.99 (0.62–1.59)	
Log-additive	–	–	–	1.26 (0.91–1.76)	0.16
Allele	A	210 (70)	234 (75)	1	0.13
	C	92 (30)	78 (25)	1.31 (0.92–1.87)	
HWE		*p* = 0.007	*p* = 0.16		
rs1801133 *(MTHFR)*					
Codominant	C/C	73 (48.3)	80 (51.3)	1	
Heterozygote	T/C	54 (35.8)	65 (41.7)	0.91 (0.56–1.47)	0.70
Homozygote	T/T	24 (15.9)	11 (7)	**2.39 (1.09–5.22)**	**0.028**
Dominant	C/C	73 (48.3)	80 (51.3)	1	0.6
	T/C-T/T	78 (51.7)	76 (48.7)	1.12 (0.72–1.76)	
Recessive	C/C-T/C	127 (84.1)	145 (93)	1	**0.017**
	T/T	24 (15.9)	11 (7)	**2.49 (1.17–5.29)**	
Overdominant	C/C-T/T	97 (64.2)	91 (58.3)	1	0.29
	T/C	54 (35.8)	65 (41.7)	0.78 (0.49–1.24)	
Log-additive	–	–	–	1.29 (0.93–1.80)	0.13
Allele	C	200 (66)	225 (72)		0.11
	T	102 (34)	87 (28)	0.76 (0.54–1.07)	
HWE		*p* = 0.013	*p* = 0.65		
rs1801131 *(MTHFR)*					
Codominant	A/A	83 (55)	86 (55.1)	1	
Heterozygote	C/A	59 (39.1)	60 (38.5)	1.02 (0.64–1.63)	0.94
Homozygote	C/C	9 (6)	10 (6.4)	0.93 (0.36–2.41)	0.88
Dominant	A/A	83 (55)	86 (55.1)	1	0.98
	C/A-C/C	68 (45)	70 (44.9)	1.01 (0.64–1.58)	
Recessive	A/A-C/A	142 (94)	146 (93.6)	1	0.87
	C/C	9 (6)	10 (6.4)	0.93 (0.37–2.34)	
Overdominant	A/A-C/C	92 (60.9)	96 (61.5)	1	0.91
	C/A	59 (39.1)	60 (38.5)	1.03 (0.65–1.62)	
Log-additive	–	–	–	0.99 (0.69–1.43)	0.97
Allele	A	225 (75)	232 (74)	1	0.97
	C	77 (25)	80 (26)	0.99 (0.69–1.42)	
HWE		*p* = 0.72	*p* = 0.91		

*n, %* number and % of individuals

^a^*OR* (Odds ratio) and 95% *CI* (confidence interval)

^b^bold values are statistically significant (*P* < 0.05), *HWE* Hardy-Weinberg equilibrium

We investigated the genotypic association between the 4 SNPs and BC risk in five genetic models including co-dominant, dominant, recessive, over-dominant and additive models. All results were age-unadjusted; the age-adjusted model (data not shown) did not diminish the significance of associations.

The AA genotype frequency of *ERCC2-*rs1799793 polymorphism revealed an association with high risk of breast cancer in both homozygote and recessive models (AA vs. GG, odds ratio (OR): 2.66, 95% confidence interval (CI): 1.20–5.91, *p* = 0.016; AA vs. GG + GA, OR: 2.90, 95% CI: 1.34–6.26, *p* = 0.0069), respectively. The association was still significant for homozygote model after Bonferrroni correction (*p* < 0,0125).

Similarly, the TT genotype of *MTHFR*-rs1801133 polymorphism was found to be associated with increased breast cancer risk in homozygote (TT vs. CC, OR: 2.39, 95% CI: 1.09–5.22, *p* = 0.028); and recessive (TT vs. CC + CT, OR: 2.49, 95% CI: 1.17–5.29, *p* = 0.017) models, but the *p* values could not withstand the Bonferroni correction. For the two remaining SNPs, no significant association was found between the ERCC2-rs13181 and *MTHFR*-rs1801131 variants and BC in any hereditary model. In addition, the allelic frequencies of all polymorphisms were similar between BC case and control groups.

### Subgroup analysis of BC cases according to age at diagnosis and family history

When BC patients were grouped into two categories with regard to age at the diagnosis (age ≤ 40 and age > 40 years) (Table 3), *MTHFR* rs1801133 revealed a positive correlation with early age at diagnosis (under 40 years); this polymorphism was found to be a BC risk factor among young patients in 4 genetic models (heterozygote: OR: 2.84, 95% CI: 1.35–6.0, *p* = 0.006; dominant: OR: 2.56, 95% CI: 1.29–5.09, *p* = 0.0061; over-dominant: OR: 2.34, 95% CI: 1.18–4.67, *p* = 0.015 and log-additive: OR: 1.62, 95% CI: 1.03–2.55, *p* = 0.036). However, the association did not meet statistical thresholds of significance after Bonferroni correction.

**Table 3:** Association of *MTHFR*-rs1801133, rs1801131, *ERCC2*-rs1799793 and rs13181 SNPs with age at diagnosis, family history, ER status, PR status and tumor size of breast cancer patients

Variable	Model	MTHFR rs1801133 Genotype	OR (95% CI)^a^	*p* ^b^	MTHFR rs1801131 Genotype	OR (95% CI)	*p*	ERCC2 rs1799793 Genotype	OR (95% CI)	*p*	ERCC2 rs13181 Genotype	OR (95% CI)	*p*
Age (Years) *(> 40 /≤40)*		C/C *(54/19)*^*c*^	1		A/A *(53/30)*	1		G/G *(46/30)*	1		A/A *(53/27)*	1	
	He	T/C *(27/27)*	**2.84 (1.35–6.00)**	**0.006**	C/A *(34/25)*	1.30 (0.66–2.57)	0.45	G/A *(35/15)*	0.66 (0.31–1.40)	0.28	C/A *(27/23)*	1.67 (0.81–3.45)	0.16
	Ho	T/T *(14/10)*	2.03 (0.77–5.33)	0.15	C/C *(8/1)*	0.47 (0.16–1.36)	0.16	A/A *(14/11)*	1.20 (0.48–3.00)	0.69	C/C *(15/6)*	0.79 (0.27–2.25)	0.37
	D	C/C *(54/19)*	1		A/A *(53/30)*	1		G/G *(46/30)*	1		A/A *(53/27)*	1	
		T/C-T/T *(41/37)*	**2.56 (1.29–5.09)**	**0.006**	C/A-C/C *(42/26)*	1.09 (0.56–2.12)	0.79	G/A-A/A *(49/26)*	0.81 (0.42–1.58)	0.54	C/A-C/C *(42/29)*	1.36 (0.70–2.63)	0.37
	R	C/C-T/C *(81/46)*	1		A/A-C/A *(87/55)*	1		G/G-G/A *(81/45)*	1		A/A-C/A *(80/50)*	1	
		T/T *(14/10)*	1.26 (0.52–3.06)	0.61	C/C *(8/1)*	0.20 (0.02–1.62)	0.07	A/A *(14/11)*	1.41 (0.59–3.37)	0.44	C/C *(15/6)*	0.64 (0.23–1.76)	0.38
	OD	C/C-T/T *(68/29)*	1		A/A-C/C *(61/31)*	1		G/G-A/A *(60/41)*	1		A/A-C/C *(68/33)*	1	
		T/C *(27/27)*	**2.34 (1.18–4.67)**	**0.015**	C/A *(34/25)*	1.45 (0.74–2.84)	0.28	G/A *(35/15)*	0.63 (0.30–1.29)	0.2	C/A *(27/23)*	1.76 (0.88–3.52)	0.11
	A	–	**1.62 (1.03–2.55)**	**0.036**	–	0.89 (0.51–1.53)	0.67	–	1.00 (0.64–1.55)	0.98	–	1.05 (0.66–1.66)	0.84
Family history *(Sporadic/Familial)*		C/C *(51/22)*	1		A/A *(41/42)*	1		G/G *(39/37)*	1		A/A *(40/40)*	1	
	He	T/C *(18/36)*	**4.64 (2.18–9.86)**	**6.8.10** ^**−5**^	C/A *(28/31)*	1.08 (0.55–2.11)	0.82	G/A *(29/21)*	0.76 (0.37–1.57)	0.46	C/A *(28/22)*	0.79 (0.39–1.60)	0.5
	Ho	T/T *(8/16)*	**4.64 (1.73–12.42)**	**0.002**	C/C *(8/1)*	0.12 (0.01–1.02)	0.05	A/A *(9/16)*	1.87 (0.74–4.76)	0.18	C/C *(9/12)*	1.33 (0.51–3.51)	0.56
	D	C/C *(51/22)*	1		A/*A (41/42)*	1		G/G *(39/37)*	1		A/A *(40/40)*	1	
		T/C-T/T *(26/52)*	**4.64 (2.33–9.21)**	**< 10** ^**−4**^	C/A-C/C *(36/32)*	0.87 (0.46–1.65)	0.66	G/A-A/A *(38/37)*	1.03 (0.54–1.94)	0.94	C/A-C/C *(37/34)*	0.92 (0.48–1.74)	0.8
	R	C/C-T/C *(69/58)*	1		A/A-C/A *(69/73)*	1		G/G-G/A *(68/58)*	1		A/A-C/A *(68/62)*	1	
		T/T *(8/16)*	2.38 (0.95–5.96)	0.057	C/C *(8/1)*	**0.12 (0.01–0.97)**	**0.01**	A/A *(9/16)*	2.08 (0.86–5.07)	0.1	C/C *(9/12)*	1.46 (0.58–3.71)	0.42
	OD	C/C-T/T *(59/38)*	1		A/A-C/C *(49/43)*	1		G/G-A/A *(48/53)*	1		A/A-C/C *(49/52)*	1	
		T/C *(18/36)*	**3.11 (1.55–6.24)**	**0.001**	C/A *(28/31)*	1.26 (0.66–2.43)	0.49	G/A *(29/21)*	0.66 (0.33–1.30)	0.22	C/A *(28/22)*	0.74 (0.37–1.46)	0.39
	A	–	**2.61 (1.60–4.25)**	**< 10** ^**−4**^	–	0.71 (0.42–1.21)	0.2	–	1.21 (0.79–1.86)	0.38	–	1.05 (0.67–1.63)	0.84
ER Status *(Positive/* *Negative)*		C/C *(52/18)*	1		A/A *(62/16)*	1		G/G *(48/22)*	1		A/A *(53/21)*	1	
	He	T/C *(35/14)*	1.16 (0.51–2.62)	0.73	C/A *(39/18)*	1.79 (0.82–3.92)	0.14	G/A *(38/12)*	0.69 (0.30–1.57)	0.37	C/A *(36/12)*	0.84 (0.37–1.92)	0.68
	Ho	T/T *(20/4)*	0.58 (0.17–1.92)	0.37	C/C *(6/2)*	1.29 (0.24–7.01)	0.8	A/A *(21/2)*	**0.21 (0.04–0.96)**	**0.045**	C/C *(18/3)*	0.42 (0.11–1.58)	0.2
	D	C/C *(52/18)*	1		A/A *(62/16)*	1		G/G *(48/22)*	1		A/A *(53/21)*	1	
		T/C-T/T *(55/18)*	0.95 (0.44–2.01)	0.88	C/A-C/C *(45/20)*	1.72 (0.80–3.69)	0.16	G/A-A/A *(59/14)*	0.52 (0.24–1.12)	0.091	C/A-C/C *(54/15)*	0.70 (0.33–1.50)	0.36
	R	C/C-T/C *(87/32)*	1		A/A-C/A *(101/34)*	1		G/G-G/A *(86/34)*	1		A/A-C/A *(89/33)*	1	
		T/T *(20/4)*	0.54 (0.17–1.71)	0.28	C/C *(6/2)*	0.99 (0.19–5.14)	0.99	A/A *(21/2)*	**0.24 (0.05–1.08)**	**0.03**	C/C *(18/3)*	0.45 (0.12–1.63)	0.19
	OD	C/C-T/T *(72/22)*	1		A/A-C/C *(68/18)*	1		G/G-A/A *(69/24)*	1		A/A-C/C *(71/24)*	1	
		T/C *(35/14)*	1.31 (0.60–2.86)	0.5	C/A *(39/18)*	1.74 (0.81–3.74)	0.15	G/A *(38/12)*	0.91 (0.41–2.02)	0.81	C/A *(36/12)*	0.99 (0.44–2.20)	0.97
	A	–	0.85 (0.50–1.42)	0.53	–	1.43 (0.78–2.65)	0.25	–	**0.54 (0.30–0.96)**	**0.028**	–	0.71 (0.41–1.23)	0.21
PR Status *(Positive/Negative)*		C/C *(46/23)*	1		A/A *(49/25)*	1		G/G *(40/27)*	1		A/A *(48/23)*	1	
	He	T/C *(27/18)*	1.33 (0.61–2.90)	0.47	C/A *(36/19)*	1.03 (0.50–2.16)	0.93	G/A *(30/17)*	0.84 (0.39–1.81)	0.66	C/A *(25/20)*	1.67 (0.77–3.61)	0.19
	Ho	T/T *(16/7)*	0.88 (0.32–2.43)	0.8	C/C *(4/4)*	1.96 (0.45–8.50)	0.4	A/A *(19/4)*	0.31 (0.09–1.02)	0.05	C/C *(16/5)*	0.81 (0.46–1.41)	0.45
	D	C/C *(46/23)*	1		A/A *(49/25)*	1		G/G *(40/27)*	1		A/A *(48/23)*	1	
		T/C-T/T *(43/25)*	1.16 (0.58–2.35)	0.67	C/A-C/C *(40/23)*	1.13 (0.56–2.28)	0.74	G/A-A/A *(49/21)*	0.63 (0.31–1.29)	0.21	C/A-C/C *(41/25)*	1.27 (0.63–2.57)	0.5
	R	C/C-T/C *(73/41)*	1		A/A-C/A *(85/44)*	1		G/G-G/A *(70/44)*	1		A/A-C/A *(73/43)*	1	
		T/T *(16/7)*	0.78 (0.30–2.05)	0.61	C/C *(4/4)*	1.93 (0.46–8.10)	0.37	A/A *(19/4)*	**0.33 (0.11–1.05)**	**0.04**	C/C *(16/5)*	0.53 (0.18–1.55)	0.23
	OD	C/C-T/T *(62/30)*	1		A/A-C/C *(53/29)*	1		G/G-A/A *(59/31)*	1		A/A-C/C *(64/28)*	1	
		T/C *(27/18)*	1.38 (0.66–2.88)	0.4	C/A *(36/19)*	0.96 (0.47–1.98)	0.92	G/A *(30/17)*	1.08 (0.52–2.25)	0.84	C/A *(25/20)*	1.83 (0.88–3.82)	0.11
	A	–	1.01 (0.63–1.61)	0.98	–	1.20 (0.68–2.14)	0.53	–	0.63 (0.38–1.04)	0.06	–	0.97 (0.60–1.57)	0.91
Tumor size *(T1 / ≥ T3)*		C/C *(10/14)*	1		A/A *(10/14)*	1		G/G *(12/16)*	1		A/A *(15/11)*	1	
	He	T/C *(11/10)*	0.65 (0.20–2.11)	0.47	C/A *(13/12)*	0.66 (0.21–2.04)	0.47	G/A *(8/8)*	0.75 (0.22–2.57)	0.65	C/A *(5/15)*	**4.09 (1.14–14.7)**	**0.03**
	Ho	T/T *(3/4)*	0.95 (0.17–5.23)	0.95	C/C *(1/2)*	1.43 (0.11–18.0)	0.78	A/A *(4/4)*	0.75 (0.16–3.62)	0.72	C/C *(4/2)*	0.68 (0.11–4.41)	0.68
	D	C/C *(10/14)*	1		A/A *(10/14)*	1		G/G *(12/16)*	1		A/A *(15/11)*	1	
		T/C-T/T *(14/14)*	0.71 (0.24–2.14)	0.55	C/A-C/C *(14/14)*	0.71 (0.24–2.14)	0.55	G/A-A/A *(12/12)*	0.75 (0.25–2.24)	0.61	C/A-C/C *(9/17)*	2.58 (0.84–7.91)	0.09
	R	C/C-T/C *(21/24)*	1		A/A-C/A *(23/26)*	1		G/G-G/A *(20/24)*	1		A/A-C/A *(20/26)*	1	
		T/T *(3/4)*	1.17 (0.23–5.82)	0.85	C/C *(1/2)*	1.77 (0.15–20.8)	0.64	A/A *(4/4)*	0.83 (0.18–3.76)	0.81	C/C *(4/2)*	0.38 (0.06–2.31)	0.28
	OD	C/C-T/T *(13/18)*	1		A/A-C/C *(11/16)*	1		G/G-A/A *(16/20)*	1		A/A-C/C *(19/13)*	1	
		T/C *(11/10)*	0.66 (0.22–2.00)	0.46	C/A *(13/12)*	0.63 (0.21–1.9)	0.42	G/A *(8/8)*	0.80 (0.25–2.6)	0.71	C/A *(5/15)*	**4.38 (1.28–15.1)**	**0.01**
	A	–	0.87 (0.40–1.91)	0.74	–	0.86 (0.34–2.15)	0.75	–	0.84 (0.40–1.76)	0.64	–	1.35 (0.60–3.03)	0.47

^a^*OR* (Odds ratio) and 95% *CI* (confidence interval)

^b^bold values are statistically significant (*p* < 0.05)

^c^number of individuals, *ER* estrogen receptor, *PR* progesterone receptor, *He* Heterozygote, *Ho* Homozygote, *D* Dominant, *R* Recessive, *OD* Overdominant, *A* Log-additive

Interestingly, in the subgroup analysis according to family history, the *MTHFR* rs1801133 showed a strong association with familial BC in almost all genetic models (heterozygote, OR: 4.64, 95% CI: 2.18–9.86, *p* = 6.8.10^− 5^; homozygote: OR: 4.64, 95% CI: 1.73–12.42, *p* = 0.002; dominant: OR: 4.64, 95% CI: 2.33–9.21, *p* < 10^− 4^; over-dominant: OR: 3.11, 95% CI: 1.55–6.24, *p* = 0.001 and log-additive: OR: 2.61, 95% CI: 1.60–4.25, *p* < 10^− 4^). These associations remained significant after Bonferroni correction (*p* < .0,005) In contrast, there was a significant association between the CC genotype of *MTHFR* rs1801131 and sporadic form of BC (recessive model: OR: 0.12, 95% CI: 0.01–0.97, *p* = 0.012). However, it turned insignificant after Bonferroni adjustment.

Otherwise, no significant association was found between the remaining polymorphisms and age at diagnosis or family history.

### Association between SNPs and BC clinicopathologic characteristics

We performed further analysis to investigate a possible relationship between the clinicopathological parameters and the distributions of SNPs genotypes in BC group. Positive results of associations are represented in Table 3.

The AA genotype of *ERCC2-*rs1799793 polymorphism was significantly frequent in ER-positive patients (homozygote model: OR: 0.21, 95% CI: 0.04–0.96, *p* = 0.045; recessive model: OR: 0.24, 95% CI: 0.05–1.08, *p* = 0.03; additive model: OR: 0.54, 95% CI: 0.30–0.96, *p* = 0.028). In addition, patients with the AA genotype of *ERCC2-*rs1799793 were prone to be PR-positive (recessive model: OR: 0.33, 95% CI: 0.11–1.05, *p* = 0.042).

Data indicated a trend to higher frequency of *ERCC2-*rs13181 CC genotype in ER-positive patients (homozygote model: OR: 0.42, 95% CI: 0.11–1.58; recessive model: OR: 0.45, 95% CI: 0.12–1.63). This increased frequency did not reach a significant level. Furthermore, patients with CA genotype showed a 1.83 fold increased risk of developing PR-negative BC (over-dominant model: OR: 1.83, 95% CI: 0.88–3.82).

Stratified analysis by tumor size feature demonstrated that *ERCC2*-rs13181 CA genotype carriers displayed an elevated risk of BC with large tumor size (T3-T4) in heterozygote model (OR: 4.09, 95% CI: 1.14–14.7, *p* = 0.03) and overdominant model (OR: 4.38, 95% CI: 1.28–15.1, *p* = 0.014).

However, these associations did not retain statistical significance after Bonferroni adjustment.

Finally, there was no evidence of significant correlation between all studied loci and other clinicopathological features including histology type, histology grade, lymph node involvement and metastasis in case subjects.

### *ERCC2* and *MTHFR* haplotype associations with BC

In this section, we performed association analysis between the risk of BC and SNP haplotypes of *ERCC2* gene (rs1799793 - rs13181) on one hand and *MTHFR* gene (rs1801133 - rs1801131) on the other hand. Haplotypes were reconstructed from the genotypic data and results of their distribution among BC cases and controls were summarized in Table 4.

**Table 4:** Association of *ERCC2* and *MTHFR* haplotypes and risk of breast cancer

Haplotype	Cases (*n* = 302)	Controls (*n* = 312)	OR (95% CI)^a^	*p* ^b^
rs1799793-rs13181 *(ERCC2)*	*n* (%)			
G-A	151 (50)	161 (51.5)	1	0.38
A-A	59 (19.5)	73 (23.5)	0.856 (0.56–1.31)	0.52
**A-C**	**41 (13.6)**	**12 (3.8)**	**3.71 (1.7–8.12)**	**0.0002**
G-C	51 (16.9)	66 (21.2)	0.82 (0.52–1.28)	0.42
**D’**	0.17	0.45		
**r**^**2**^	0.03	0.02		
rs1801133-rs1801131 *(MTHFR)*	*n* (%)			
C-A	146 (48.3)	164 (52.5)	1	0.26
T-A	79 (26.2)	68 (21.9)	1.3 (0.84–2.03)	0.17
T-C	23 (7.5)	19 (6)	1.36 (0.63–2.96)	0.39
C-C	54 (18)	61 (19.6)	0.99 (0.61–1.62)	0.59
**D’**	0.12	0.16		
**r**^**2**^	0.003	0.003		

*N* (%): number and % of haplotypes

^a^*OR* (Odds ratio) and 95% *CI* (confidence interval)

^b^bold values are statistically significant (*p* < 0.05), *D’* Lewontin’s standardized disequilibrium coefficient, *r*^*2*^ squared correlation coefficient

The pairwise linkage disequilibrium is given for each pair of SNPs. The observed low D’ values (0.17 in cases and 0.45 in controls) and low r^2^ (0.03 in cases and 0.02 in controls) indicated that the studied *ERCC2* SNPs were not at high linkage disequilibrium. Likewise, *MTHFR* were not found in linkage disequilibrium in both cases and controls (cases: D’ = 0.12, r^2^ = 0.03; controls: D’ = 0.16, r^2^ = 0.003).

For *ERCC2* SNPs, we found all the four expected haplotypes in both cases and controls; the most popular haplotype was G-A, followed by haplotypes A-A, G-C and A-C (cases: 50, 19.5, 16.9 and 13.6%; controls: 51.5, 23.5, 21.2 and 3.8%, respectively). The haplotype containing the two minor alleles A-C was distributed differently between patients and controls. It was significantly associated with about 3.71 fold increase risk of BC when compared to the wild-type haplotype G-A (OR: 3.71, 95% CI: 1.7–8.12, *p* = 0.0002). These association was maintained after Bonferroni correction (*p* < 0,0125). The three other haplotypes were distributed similarly between case and control groups.

Considering *MTHFR* gene, all the four expected haplotypes appeared in our analysis. The most frequent for both BC cases and controls was C-A (rs1801133 C - rs1801131 A) haplotype (48.4 and 52.5%, respectively). The estimated frequencies for the other haplotype were: T-A (26.2 and 21.9%), C-C (18 and 19.6%) and T-C (7.5 and 6%) in cases and controls, respectively. No difference was observed between case and control groups regarding the distribution of all the haplotypes. These findings indicated no statistically significant associations of *MTHFR* haplotypes with BC risk.

We also considered the association between haplotypes and major clinicopathological features of BC patients. As presented in Table 5, our data suggested the haplotype composed of the 2 alternative alleles of *MTHFR* SNPs (rs1801133/T - rs1801131/C) was strongly associated with early age at diagnosis of BC (15.4% of early onset BC vs. 5.1% diagnosed after 40 years; OR: 3.52, 95% CI: 1.23–10.06, *p* = 0.02). It was also found to be significantly more frequent in familial forms of BC than in sporadic (OR: 4.96, 95% CI: 1.5–16.42, *p* = 0.0097). Contrariwise, the haplotype with one minor allele rs1801131/C (C-C) was more frequent in sporadic BC than familial (24.5% vs. 7.4%; OR: 0.36, 95% CI: 0.14–0.95, *p* = 0.042). However, none of these associations reached the Bonferroni-adjusted significance threshold (*p* > 0,005).

**Table 5:** Association of *ERCC2* and *MTHFR* haplotypes with clinical characteristics of breast cancer patients

Haplotype (*n* = 302)	Age (years)		OR (95% CI)^a^	*p* ^b^
	> 40	≤40		
rs1801133-rs1801131				
C-A	49.8% ^c^	49.4%	1	–
T-A	23.9%	26.5%	1.08 (0.61–1.93)	0.78
C-C	21.2%	8.7%	0.44 (0.16–1.16)	0.1
T-C	**5.1%**	**15.4%**	**3.52 (1.23–10.06)**	**0.02**
rs1799793-rs13181				
G-A	51.3%	48.1%	1	–
A-A	18.7%	20.7%	1.23 (0.65–2.35)	0.53
G-C	15.5%	18.9%	1.33 (0.68–2.61)	0.41
A-C	14.5%	12.3%	0.91 (0.47–1.76)	0.79
Haplotype	Family history of breast cancer			
(*n* = 302)	Sporadic	Familial		
rs1801133-rs1801131				
C-A	53.5%	46.6%	1	–
T-A	17.9%	31.1%	1.76 (0.97–3.18)	0.063
C-C	**24.5%**	**7.4%**	**0.36 (0.14–0.95)**	**0.042**
T-C	**4.1%**	**14.9%**	**4.96 (1.50–16.42)**	**0.0097**
rs1799793-rs13181				
G-A	51.1%	48.7%	1	–
A-A	19.1%	20.2%	1.05 (0.56–1.98)	0.88
G-C	18.4%	15.5%	0.87 (0.44–1.68)	0.67
A-C	11.4%	15.6%	1.32 (0.70–2.47)	0.39
Haplotype	ER and PR status			
(*n* = 286)	ER positive	ER negative		
rs1799793-rs13181				
G-A	46.7%	57.6%	1	–
A-A	19.6%	17.4%	0.83 (0.38–1.81)	0.65
G-C	15.9%	20.2%	1.11 (0.54–2.30)	0.78
A-C	**17.8%**	**4.8%**	**0.28 (0.09–0.88)**	**0.03**
rs1799793-rs13181	PR positive	PR negative		
G-A	48.7%	48.3%	1	–
A-A	19.3%	20.5%	1.41 (0.67–2.98)	0.36
G-C	**13.1%**	**25.7%**	**2.34 (1.1–4.96)**	**0.02**
A-C	**18.9%**	**5.6%**	**0.33 (0.12–0.92)**	**0.03**
Haplotype	Tumor size			
(*n* = 72)	T1	T4		
rs1799793-rs13181				
G-A	55.5%	41.7%	1	–
A-A	17.4%	29.2%	2.10 (0.59–7.46)	0.26
G-C	**11.2%**	**29.1%**	**23.30 (2.03–267.69)**	**0.017**
A-C	15.9%	0%	NA	1

^a^*OR* (Odds ratio) and 95% *CI* (confidence interval)

^b^bold values are statistically significant (*p* < 0.05), *n* number of halotypes

^c^% of haplotypes, ER: estrogen receptor, *PR* progesterone receptor, *NA* not applicable

With respect to ERCC2 gene, the haplotype composed of the 2 minor alleles (rs1799793/A - rs13181/C) was significantly associated with positive ER and PR expressions in BC tumors (ER: OR: 0.28, 95% CI: 0.09–0.88, *p* = 0.03; PR: OR: 0.33, 95% CI: 0.12–0.92, *p* = 0.017). However, patients who carried G-C haplotype tended to be more likely to develop BC with negative-PR tumors (OR: 2.34, 95% CI: 1.1–4.96, *p* = 0.02) and with large tumor size, T4 (OR: 23.3, 95% CI: 2.03–267.69, *p* = 0.017) than did positive PR patients and T1 tumor size ones, respectively. No statistical associations were detected after Bonferroni correction.

Finally, we did not discover any association with *MTHFR*, *ERCC2* haplotypes and other clinicopathological parameters of BC.

## Discussion

The etiology of breast cancer is complex and multifactorial, as sustained by contribution of various environmental and genetic factors. Beside mutations in predisposition genes, the identification of genetic polymorphisms including SNPs in the genes conferring relatively small increment in BC risk could be beneficial for the understanding of the disease mechanisms. Such information could also be of great interest in identifying high risk individuals and in improving cancer prevention strategies.

Accordingly, in our present case-control study, we investigated whether 4 SNPs of *ERCC2* and *MTHFR* genes affect the pathogenesis of BC in Moroccan population.

The results from this study revealed, for the first time, an association of the four polymorphisms with increased risk of breast cancer and/or with disease sub-phenotypes including age at diagnosis, family history, hormone receptor statuses and tumor size in Moroccan BC patients.

The first polymorphism, i.e., *ERCC2-*rs1799793 was identified as potential risk factor for BC in this work. Indeed, homozygote carriers of the minor allele (A/A genotype, Asn312Asn) were over-represented in the BC cases compared to controls, which would make them at high risk of developing the disease among Moroccan cases.

The variant *ERCC2-*rs1799793 is a G > A coding polymorphism causing a codon 312 Asp to Asn amino acid exchange in *ERCC2* gene. *ERCC2* is an essential gene involved in DNA damage repair pathway and whose product, a DNA helicase, is important in the transcription-coupled nucleotide excision repair process that contribute to preserving integrity and stability of the genome. It is well known that the DNA repair ability is an important determinant of the predisposition toward various malignancies [9, 24, 25]. There is increasing data supporting the hypothesis that genetic polymorphisms (SNPs) in DNA repair genes could lead to disorder in DNA repair machinery resulting in accumulation of mutations, and in turn could contribute to increased susceptibility to various types of cancers including BC [17, 25–27]. In particular, the functional SNPs *ERCC2*-rs1799793 and *ERCC2*-rs13181 enrolled in our study have been previously associated with specific DNA defects, namely defective repair capacity of ultraviolet light-induced DNA damage [28]. Interestingly, data from a study conducted by wolf et al. [29] indicated that both polymorphisms significantly decreased constitutive *ERCC2* mRNA levels in lymphocytes of healthy subjects which consequently reduce *ERCC2* protein amounts.

Our findings revealed an association of *ERCC2*-rs1799793/AA genotype with increased risk of BC. These results are corroborated by previous studies in various populations, such as Russians, Mexicans, Chinese, Egyptian, and Taiwanese [17, 30–33]. However, the results were not unanimous as other studies of this SNP failed to find positive correlation with BC, especially among Caucasians as well as North American, European sub-populations, Chinese, Portuguese, Poland and Australian [9, 34–39]. At the opposite, the recessive genotype was reported to be protective in Asian and Chinese populations [9, 33, 36, 40–42]. All of these studies agree to take into account ethnic origin, sample characteristics and environmental factors that interact with that variant in the reading of these results.

The frequency of *ERCC2*-rs1799793 minor allele (A) reported in our Moroccan control group was 27%. According to 1000 Genomes Project Phase 3 data [43], this value was higher than in East-Asian and African American (5 and 10%, respectively) and lower than that of South Asian and European (34 and 36%, respectively).

The analysis of association between *ERCC2*-rs1799793 and clinicopathological features showed that women cases with homozygote AA genotype were more likely to have ER-positive and PR-positive breast cancer compared with women carrying the GG genotype. It is believed that an over-expression of ER in BC could be involved in the tumorogenesis by stimulating mammary cells proliferation which leads to uncontrolled cell division and accumulation of DNA mutations. Therefore, one of the therapeutic means relies on the use of ER modulators [44]. Our results suggested that *ERCC2*-rs1799793 could be a potential risk marker for hormone receptor-positive BC in Moroccan population. Otherwise, inconsistent findings were reported in a prior study showing that Chinese women with heterozygous genotype were more prone to develop PR-negative BC compared to wild type genotype [9].

In the current study, we included the coding SNP, *ERCC2*-rs13181 due to its functional relevance [28]. This polymorphism changes the charge of the amino acid (nucleotide A to C substitution causing a 751 Lys to Gln amino acid change) and is located in a crucial domain of interaction between *ERCC2* protein and p44, its helicase activator within the transcription factor TFIIH complex [45]. Despite the overrepresentation of the alternative homozygote genotype CC (Gln751Gln) in subgroup of Moroccan BC patients (OR: 1.84, 95% CI: 0.86–3.91) and which did not reach significant levels, the results may indicate a possible association with BC risk in view of HWE results. Indeed, there was a clear deviation from the HWE in BC subjects for both SNPs (*p* = 0.007 for *ERCC2*-rs13181, and *p* = 0.002 for *ERCC2*-rs1799793), while there was an accordance with HWE in controls (*p* = 0.16 and 0.52, respectively). This indicated that no evolutionary change has occurred affecting the distribution of the normal and alternative alleles in general population. Tupikowski et al. [46] reported similar situation on renal cell carcinoma. Some authors claimed that screening with HWE datasets of affected individuals is relatively efficient to detect genes associated to a disease [46, 47].

In our study, it is possible that the influence of *ERCC2*-rs13181 CC genotype might be masked by the small size of tested groups. Thus, larger studies are warranted to reveal associations that are not immediately apparent.

As reported by other study populations, there is mixed evidence regarding the contribution of *ERCC2*-rs13181 polymorphism to the risk of BC. Significant associations with increased risk of BC were found in some populations such as Caucasians, African Americans and Indians [35, 36, 41, 48, 49]. At the opposite, other reports stated that there was no evidence of association for populations of China, North America and Europe [17, 30, 33, 34, 36, 38, 40]. These findings suggested, again, a possible role of the environment, ethnic differences and variable genetics backgrounds in cancer development.

The *ERCC2*-rs13181 minor allele frequency reported in our study in Moroccan controls (25%) was slightly higher than American (21.5%) and lower than Europeean and Asian frequencies (36.4 and 34.7%, respectively) [43].

In regard to clinicopathological variables, our study showed that *ERCC2*-rs13181 heterozygote genotype was more prevalent in the BC patients with higher tumor size T3-T4 which is a poor prognostic indicator. These results suggested that *ERCC2*-rs13181 is more associated with the severity of the disease than its risk and may serve as a biomarker for BC progression in Moroccan population.

In the analysis of association between *ERCC2*-(rs1799793-rs13181) haplotypes and the risk of BC, we inferred that the haplotype defined by the minor alleles A-C may play a substantial role in increasing the risk of BC. Interestingly, the level of significance was higher (*p* = 0.0002) than it was when the two SNPs were taken individually (*p* = 0.0069 for *ERCC2*-rs1799793 and *p* = 0.11 for *ERCC2*-rs13181). This result supports a potential correlation of *ERCC2*-rs13181 with increased BC risk in Moroccan patients in addition to that of *ERCC2*-rs1799793. This haplotype may be regarded as susceptibility marker to BC in Moroccan patients. However, this result differed from previous studies. Indeed, the same haplotype was associated with marginal risk of BC in North-Eastern Poland population [38], but failed to exert any effect in African Americans [50]. In the latter population, the haplotype defined by major alleles (G-A) was found more frequently among controls than cases [50], while the G-C combination was considered as the most potent risk-conferring haplotype in German population [51].

Otherwise, it appears that these two polymorphisms have low linkage disequilibrium in both Moroccan cases and controls suggesting that they are located in a haplotype block with high rate of recombination between the two loci. These two SNPs could therefore be regarded as two distinct hereditary units. Previous studies have reported similar results in populations of European and African ancestry based on the HapMap data [50], in contrast to the US and Poland populations where they are in linkage disequilibrium [38, 52]. In our study, the A-C haplotype was found to correlate with ER+ and PR+ expression, whereas the G-C haplotype was connected with higher risk of developing a PR negative and high tumor size BC in Moroccan cases. Accordingly, these two haplotypes could be considered as markers of breast cancer prognosis.

The other most relevant result of the current work was the association of *MTHFR*- rs1801133 (C677T) polymorphism with increased susceptibility for BC in Moroccan patients. We have detected more homozygote carriers (TT) among patients than controls. The genotype distributions of *MTHFR*-rs1801133 had a somewhat deviation from the HWE in BC cases, but not in healthy controls, giving further evidence of its role in increased BC risk.

MTHFR is the gene encoding methylenetetrahydrofolate reductase enzyme which is involved in folate metabolism. The enzyme assists the irreversible conversion of 5,10-methylenetetrahydrofolate (5,10-MTHF) to 5-methyltetrahydrofolate (5-MTHF). The 5-MTHF, the predominant circulatory form of folate, plays an integral role in DNA synthesis, DNA methylation and DNA repair and maintenance. Deficiency of folate has been shown to result in DNA damage, DNA hypomethylation and reduced DNA repair leading to an increased risk of chromosomal breaks. It has been appreciated that depletion of folate might be linked to the carcinogenesis in multiple cancer conditions through the process cited above. Consequently, the potential influence of MTHFR activity on folate availability makes the MTHFR gene an attractive candidate for cancer predisposition [53].

Accordingly, the most common studied functional MTHFR variants, rs1801133/C677T (Ala222Val) and rs1801131/A1298C (Glu429Ala) have been shown to generate therm-olabile and less active enzyme, with C677T having a higher effect than A1298C variant [54–56].

In the case of breast cancer, rs1801133 and rs1801131 have been widely assessed for their implication in increasing breast cancer risk in numerous epidemiological studies. However, conflicting results have been reported depending on the study group. For several studies, there was a significant association between rs1801133 SNP and high risk of BC [53, 57–61], whereas a number of others failed to detect any association [62–68].

When taking the age at diagnosis and family history into account, we found a strong association of *MTHFR*-rs1801133 with young age (< 40 years) and with familial form of BC in Moroccan patients. Campbell et al. [69] and Semenza et al. [70] reported similar results of significant association between *MTHFR*-rs1801133 variant and early onset of breast cancer (before 40 years of age) in English population. Another study showed that the risk estimates were maintained in group of women diagnosed at or before 50 years [71]. Early age of diagnosis is well accepted as a prognostic indicator associated to more aggressive form of BC [72]. Moreover, family history is a particularly major factor associated with increased risk of BC. Our finding are in accordance to an earlier study conducted in Jewish population showing high frequency of hereditary BC in individuals with TT genotype [73] and in Italian *BRCA1* mutations carriers [74]. Therefore, our results suggested *MTHFR*-rs1801133 polymorphism as a real risk modifier in overall Moroccan BC cases, especially in young and familial subgroups.

Otherwise, the *MTHFR*-rs1801133 had no statistically significant association with clinicopathologic features. This result was supported by a recent report in Indian subjects [59] and by previous findings in Brazilian and Austrian cases. Meanwhile Huang et al. [75] reported only a weak correlation of *MTHFR* C/T or TT genotype distribution with RE positive status in Taiwanese population.

The frequency of the *MTHFR*-rs1801133 variant allele amongst healthy Moroccan population (28%) was similar to East-Asian one (29.6%) but lower than those reported in American (47.4%) and European (36.5%) [43].

Regarding the second SNP, *MTHFR*-rs1801131, while there was no significant association with overall BC risk in Moroccan patients, this polymorphism could be a potential marker for sporadic BC subphenotype and marginally for patients aged over 40 years. Similarly, high risk of sporadic breast cancer associated with *MTHFR*-rs1801131 was previously reported in Turkish women carrying homozygote variant genotype [76]. Likewise, the presence of the alternative C allele confered an increased risk of breast cancer in sporadic cases of Italian population [74].

In the light of these findings, and although both *MTHFR* polymorphisms affect the total enzymatic activity, it is not excluded that their dissimilar contributions in modulating breast cancer risk direction and extend may depend on interactions with other still unknown endogenous or exogenous factors.

We further evaluated the contribution of *MTHFR* haplotypes generated by *MTHFR*-rs1801133 and *MTHFR*-rs1801131 SNPs to BC risk. The current findings displayed a frequency of 6 and 7.5% for the haplotype T-C in Moroccan healthy population and in BC cases. Different data were reported in previous studies depending on ethnic origin. The estimated frequency was reported to be zero in German, Spanish and Japanese populations [67, 77, 78]. Nevertheless, a recent Arabic study showed slightly higher frequency in Jordanian population (8.3%) and lower frequency (3.6%) in matched BC cases [79].

Haplotype analysis inferred that none of the *MTHFR* haplotypes was significantly associated with overall BC risk in our population, although we have noticed that carriers of the haplotype defined by the minor alleles (T-C) were 1.36 times more likely to have BC. However, this haplotype exhibited a positive correlation with familial form and with early onset of the disease. At the opposite, the C-C haplotype showed higher representation in sporadic BC subgroup. Other clinical conditions of BC were independent of *MTHFR* haplotypes in Moroccan patients.

Previous studies that investigated the contributory role of *MTHFR* haplotypes in BC development have produced inconclusive results. A borderline line significant protection was observed for the C-C haplotype in German and East asian populations [67, 80], while the C-A haplotype was protective in South-Eastern European population [54]. Carriers of the T-C haplotype in Caucasians [80] and of the T-A haplotype in Jordanian [79] were more prone to develop BC.

Interestingly, the Lewontin’s estimate was consistent with no linkage disequilibrium in both cases and controls, suggesting that both SNP were independent of each other. It seems likely that there is a specific linkage disequilibrium pattern in Moroccan population for these *MTHFR* SNPs, suggesting that they act independently to affect BC susceptibility. LD patterns at these loci appear to be population dependant. LD was strong in populations of Europe, Brazil, Pakistan and china [78, 81, 82] and much smaller in Mexican and African populations [78]. In contrast, these two variants are genetically independent in Russian and Puerto Rican populations [82, 83].

## Conclusion

To the best of our knowledge, this is the first study assigning increased BC risk to *ERCC2*-rs1799793 (Asn312Asn) polymorphism and the corresponding haplotype determined by Asn312-Gln751 codons in Moroccan population.

The other finding of special interest is the association between *MTHFR*-rs1801133 (Val222Val) with increased risk of BC. Our results suggested that *ERCC2*-rs1799793 and MTHFR-rs1801133 represent suitable tool for assessing susceptibility to breast cancer in Moroccan population and prognosis.

For the two other SNPs investigated in this study, it is likely that either they do not contribute to BC risk or, more likely, their influence is small and can be detected only in larger samples. Thus, it is strongly recommended to reproduce these results on a larger number of participants.

## Abbreviations

BC: Breast cancer
CI: Confidence interval
ER: Estrogen receptor
FH: Family history
Her-2: Human epidermal growth factor receptor 2
HWE: Hardy-Weinberg equilibrium
LD: Linkage disequilibrium
OR: Odds-ratio
PR: Progesterone receptor
SBR: Scarff-Bloom-Richardson
SNP: Single nucleotide polymorphism
